# Reply to: Questioning the role of HIPEC in patients with granulosa cell ovarian tumours

**DOI:** 10.1515/pp-2024-0025

**Published:** 2024-12-05

**Authors:** Luis Felipe Falla-Zuniga, Armando Sardi, Mary Caitlin King, Vadim Gushchin

**Affiliations:** Surgical Oncology, 158303 Mercy Medical Center, Baltimore, MD, USA

To the Editor:

We thank Drs. Iavazzo and Gkegkes for their knowledgeable comments on our study [1]. We gladly provide further information on our patients with granulosa cell tumors. At the time of publication, three patients with granulosa cell tumors had undergone cytoreductive surgery and hyperthermic intraperitoneal chemotherapy (CRS/HIPEC) at our institution. As described in our manuscript in Table 1 [2], all three patients underwent CRS/HIPEC for recurrent disease. Time from diagnosis to CRS/HIPEC ranged from 24 to 138 months. One patient underwent CRS/HIPEC after an initial CRS. In the remaining two patients, CRS/HIPEC was performed after previous local surgical resection (salpingo-oophorectomy) and platinum-based adjuvant chemotherapy. Time from initial management to first recurrence ranged from to 12.9 to 133.7 months. No information was provided regarding adjuvant chemotherapy or first site of recurrence after CRS/HIPEC because none had received adjuvant chemotherapy or recurred at the time of the publication.

Regarding survival outcomes, progression-free survival (PFS) was calculated from the date of CRS/HIPEC to recurrence, death, or last follow-up. Overall survival (OS) was measured from the date of CRS/HIPEC to death or last follow-up. Given that one patient died of complications before disease recurrence, and the two others were disease free at the time of last follow-up, PFS and OS were virtually the same at the time of publication. Due to the limited sample size, Kaplan–Meier curves for each histology were not possible.

As some time has passed since the publication, we can provide updated survival and follow up information for the granulosa cell tumor patients listed in Table 1. Currently, one patient (Table 1; patient ID: 11) did not receive any adjuvant treatment and remains with no evidence of disease 58.7 months after CRS/HIPEC. The other patient (ID 12) experienced intra-abdominal recurrence (spleen, left upper quadrant, and pelvis) at 10.9 months postoperatively. Recurrence was treated with a second complete CRS/HIPEC with cisplatin + doxorubicin. The patient remained disease-free for 10.4 months, recurring within the lower abdominal wall. After a wide local excision, the patient is currently disease-free with an OS from first CRS/HIPEC of 33.4 months. This patient did not receive adjuvant chemotherapy after any of the surgical procedures. As mentioned above, the third patient (ID 13) died at 0.5 months due to postoperative septic shock secondary to ischemic bowel disease without receiving adjuvant therapy.

Current clinical guidelines recommend considering secondary CRS in patients with recurrent granulosa cell tumors, always aiming for a complete tumor resection. PFS after secondary CRS has been reported 39–57 months [3, 4] and, as mentioned by Iavazzo and Gkegkes [1], the use of adjuvant chemotherapy has not improved survival [3]. We now report an updated PFS of 11 and 59 months, which is in line with other studies summarized by Yasukawa et al. (PFS ranging from 25 months to 19 years) [5]. Given the mixed results, agents, and protocols used, further investigations are needed. However, we consider complete CRS/HIPEC as a feasible therapeutic option in recurrent granulosa cell tumors.
